# Imatinib prevents blood-spinal cord barrier disruption by inhibiting PDGFR-mediated JMJD3 expression and activation after spinal cord injury

**DOI:** 10.1186/s12987-025-00690-5

**Published:** 2025-07-16

**Authors:** Chan Sol Park, Jee Youn Lee, Tae Young Yune

**Affiliations:** 1 Age-Related and Brain Diseases Research Center, Kyung Hee University, Seoul, 02447 Republic of Korea; 2 Department of Biomedical Science, Kyung Hee University, Seoul, 02447 Republic of Korea; 3 Department of Biochemistry and Molecular Biology, School of Medicine, Kyung Hee University, Seoul, 02447 Republic of Korea; 4 KHU-KIST Department of Converging Science and Technology, Kyung Hee University, Seoul, 02447 Republic of Korea; 5 Department of Biochemistry and Molecular Biology, and Age-Related and Brain Diseases Research Center, School of Medicine, Kyung Hee University, Medical Building 10th Floor, 26, Kyungheedae-ro, Dongdaemun-gu, Seoul, 02447 Republic of Korea

**Keywords:** Spinal cord injury, Blood-spinal cord barrier, Imatinib, JMJD3, MMP, PDGFR

## Abstract

**Background:**

After a spinal cord injury (SCI), disruption of the blood-spinal cord barrier (BSCB) leads to secondary injuries, including inflammatory responses and apoptotic cell death, ultimately causing permanent neurological deficits. Imatinib, a tyrosine kinase inhibitor, has been reported to enhance BSCB integrity and improve functional recovery after SCI. However, the mechanism by which imatinib regulates BSCB integrity remains unclear. Recent studies have identified the histone H3K27me3 demethylase JMJD3 as a key mediator of BSCB disruption, with high expression observed in blood vessels after SCI. In this study, we investigated whether imatinib regulates JMJD3 expression and activation through PDGFR signaling, thereby mitigating BSCB disruption following SCI.

**Methods:**

Imatinib (100 mg/kg) was administered intraperitoneally to rats subjected to a contusion injury at the T9 level of the spinal cord and was continued daily for 14 days.

**Results:**

Our results indicate that imatinib inhibited the phosphorylation of PDGFRα and PDGFRβ, both tyrosine kinase receptors, without affecting their expression levels. Additionally, imatinib reduced JMJD3 and MMP-9 expression and activation in blood vessels, thereby decreasing macrophage infiltration after SCI. In an oxygen-glucose deprivation (OGD)-induced bEnd.3 cell model, phosphorylated PDGFRα and PDGFRβ, along with JMJD3 expression and activation, were significantly upregulated but were effectively inhibited by imatinib treatment. Furthermore, imatinib suppressed secondary damage, including cell death, blood cell infiltration (e.g., neutrophils and macrophages), inflammation, axonal and myelin loss, and lesion volume. These effects collectively resulted in significant improvements in functional recovery after SCI.

**Conclusion:**

Based on these findings, we propose that imatinib exerts a neuroprotective effect, in part by inhibiting PDGFR-mediated JMJD3 expression and activation following SCI.

**Supplementary Information:**

The online version contains supplementary material available at 10.1186/s12987-025-00690-5.

## Background

Spinal cord injury (SCI) leads to motor and sensory impairments, which result from both primary and secondary injuries [1, 2]. The primary injury occurs immediately after the initial trauma, causing structural failure. This is followed by secondary injuries, including disruption of the blood-spinal cord barrier (BSCB), inflammation, and apoptotic cell death, which ultimately result in progressive and permanent functional deficits [3, 4].

The BSCB plays a critical role in preventing the entry of circulating toxins and pathogens into the spinal cord [5]. SCI-induced disruption of the BSCB contributes to secondary damage by allowing the influx of endogenous inflammatory factors and pathogens into spinal cord tissue. In particular, increased BSCB permeability leads to continuous infiltration of inflammatory cells into the spinal cord. This triggers neuroinflammation in the spinal parenchyma, where activated inflammatory cells produce elevated levels of inflammatory mediators [5]. Consequently, BSCB disruption and damage are central to the progression of secondary injury following SCI.

Several studies have demonstrated that matrix metalloproteinases (MMPs), enzymes responsible for degrading the extracellular matrix and modulating cytokines and growth factors, become abnormally and excessively activated following central nervous system (CNS) injury. This excessive activation degrades tight junction (TJ) components in endothelial cells, leading to disruption of the blood-brain barrier (B-BB) or BSCB after ischemic brain injury and SCI [6–8]. Recently, we reported that inhibiting MMP-9 expression and activity after SCI using gallic acid, fluoxetine, valproic acid, carvacrol, and protocatechuic acid effectively reduces BSCB disruption and subsequent blood cell infiltration, inflammation, and cell death, resulting in improved functional recovery [7, 9–12]. Our findings also demonstrated that the histone H3K27me3 demethylase JMJD3 is upregulated in damaged brain and spinal cord vasculature and plays a critical role in B-BB or BSCB disruption following CNS injury by directly regulating MMP gene expression [7, 11, 13, 14]. Thus, targeting JMJD3 may represent a promising therapeutic strategy to prevent secondary injuries after SCI, although the mechanism driving JMJD3 upregulation after SCI remains unclear.

Platelet-derived growth factor receptor (PDGFR), a receptor tyrosine kinase, is a critical regulator of cell proliferation, growth, and the progression of CNS diseases, including ischemic stroke and SCI. PDGFR consists of two main isoforms, PDGFRα and PDGFRβ, and is expressed in various CNS cell types, including pericytes, astrocytes, oligodendrocyte precursor cells, microglia, and neurons [15]. Upon auto-phosphorylation, PDGFR initiates a cascade of intracellular signaling events. Phosphorylated tyrosine residues on activated receptors serve as docking sites for signaling molecules, leading to the activation of downstream pathways, including PI3K/AKT, STAT, and MAPK pathways [16]. Recent studies have highlighted diverse roles of PDGFR in vascular biology, including its regulation of B-BB integrity. For example, imatinib, a PDGFR inhibitor, has been shown to normalize B-BB integrity following ischemic stroke and subarachnoid hemorrhage [17–20]. Additionally, PDGFRβ signaling plays a crucial role in stabilizing blood vessels; inhibition of PDGFRβ signaling has been associated with increased B-BB permeability and aggravated brain edema in a mouse model of cerebral ischemia [21]. Furthermore, imatinib has been reported to enhance BSCB integrity and improve functional outcomes after SCI [22]. However, the mechanism by which PDGFR inhibition by imatinib regulates BSCB integrity remains unknown.

In this study, we investigated whether imatinib prevents BSCB disruption by regulating PDGFR-mediated JMJD3 expression and activation, followed by the downregulation of MMP gene expression after SCI.

## Materials and methods

### Spinal cord injury

Adult male Sprague–Dawley rats (240–260 g; Samtako, Osan, Korea) were used in this study. SCI was induced using the NYU impactor, following previously described methods [23]. Rats were anesthetized with an intraperitoneal (i.p.) injection of chloral hydrate (500 mg/kg), and a laminectomy was performed at the T9-T10 level to expose the spinal cord without disrupting the dura. The spinous processes of T8 and T11 were clamped to stabilize the spine, and a moderate contusion injury (10 g × 25 mm) was applied to the dorsal surface of the exposed spinal cord using the NYU impactor. Body temperature was maintained at 37 ± 0.5 °C throughout the surgical procedure using a heating pad (Biomed S.L., Alicante, Spain). Following the injury, the muscles and skin were sutured in layers, and the rats were placed in a controlled chamber overnight under optimal temperature and humidity conditions. Post-surgery, the rats received additional fluids (5 ml of lactated Ringer’s solution) and antibiotics (gentamicin, 5 mg/kg, i.m.) once daily for 5 days. The bladder was manually emptied three times daily until the rats regained reflexive bladder control. All animal experiments were conducted in accordance with the guidelines of the Animal Care Committee of Kyung Hee University (permission number: KHSASP-19-096).

### Drug administration

Rats were randomly divided into three groups: the sham-operated control group, the SCI vehicle group, and the imatinib-treated group. In the sham control group, rats underwent a laminectomy at the T9-T10 level without a contusion injury and did not receive any pharmacological treatment during the experiment. Based on our preliminary study, we found that a dose of 100 mg/kg of imatinib (Novartis, Switzerland) and 10 µg of GSK-J4 (Merck, Darmstadt, Germany) was an optimal dose for the reduction of BSCB disruption after SCI (Supplementary Figs. 1 and 2) and thus we used 100 mg/kg of imatinib and 10 µg of GSK-J4 throughout this study. Imatinib was dissolved in 0.1 M PBS (pH 7.4) and administered intraperitoneally at a dosage of 100 mg/kg immediately after SCI and again 8 h later. Subsequently, the same dosage was administered once daily for 14 days for behavioral testing and other experiments at specified time points. The vehicle group received an equivalent volume of 0.1 M PBS via intraperitoneal injection. For certain experiments, GSK-J4 (10 µg), an inhibitor of the histone demethylase JMJD3/UTX, was dissolved in 50% dimethyl sulfoxide (DMSO) in saline. The solution was administered via intraspinal injection at a volume of 2 µl immediately after SCI. The intraspinal injection procedure was performed as previously described [24]. Briefly, a pulled glass capillary micropipette attached to a Hamilton syringe, connected to a micromanipulator, was used to deliver the solution into the lesion epicenter at a depth of 1.5 mm. The injection rate was maintained at 0.5 µl/min to ensure precise administration. The vehicle group received an equivalent volume of 50% DMSO in saline. No significant side effects, such as changes in body weight or increased mortality, were observed throughout the experimental period.

### bEnd.3 cell culture and oxygen-glucose deprivation/reperfusion (OGD/R) injury

A mouse brain endothelial cell line, bEnd.3, obtained from the American Type Culture Collection (Manassas, VA), was cultured as described in previous studies [7, 25]. Briefly, bEnd.3 cells were grown in Dulbecco’s Modified Eagle Medium (DMEM; Sigma-Aldrich, St. Louis, MO) supplemented with 10% FBS until confluent. The culture medium was then replaced with glucose-free DMEM (Sigma), and the cells were transferred to a humidified anaerobic chamber (0.1% O₂, 5% CO₂, and 94.9% N₂) (APM-30D, Astec, Fukuoka, Japan), following the protocol described by Park et al. [11]. After 6 h of oxygen-glucose deprivation (OGD), the cells were returned to normoxic conditions and cultured in DMEM containing 25 mM glucose to initiate reoxygenation. Control cells were cultured in DMEM with 25 mM glucose under normoxic conditions. Imatinib (10 µM) and GSK-J4 (4 µM) were dissolved in 0.1 M PBS and DMSO, respectively, and applied 30 min before OGD/R injury. The vehicle control group received an equivalent volume of 0.1 M PBS and DMSO.

### Measurement of transendothelial electrical resistance

Endothelial permeability was evaluated by measuring transendothelial electrical resistance (TEER) according to the manufacturer’s instructions (Millipore, Billerica, MA) and as described in previous studies [11, 13]. After cells were subjected to OGD/R injury, TEER was measured using a Millicell ERS-2 V-Ohm Meter (Millipore). Resistance values were recorded in ohms (Ω), and TEER was calculated by multiplying the measured resistance by the surface area of the transwell inserts, expressed as ohms per square centimeter (Ω·cm²).

### Evans blue assay

The Evans Blue dye extravasation assay was used to assess BSCB disruption, following the method described by Lee et al. [9]. Briefly, 5 ml of a 2% Evans Blue dye solution (Sigma) in 0.9% saline was administered intraperitoneally. After three hours, the rats were sacrificed by intracardiac perfusion with 0.1 M PBS. A 5 mm segment of the T9 spinal cord, centered on the lesion site, was carefully removed and homogenized in 50% trichloroacetic acid solution. The concentration of Evans Blue in the homogenate was measured using a spectrophotometer (Molecular Devices, Sunnyvale, CA) at wavelengths of 620 nm (excitation) and 680 nm (emission). The dye concentration was quantified using a standard curve constructed with known quantities of Evans Blue and expressed as micrograms per gram of tissue (µg/g).

### Tissue preparation

At the indicated time points, rats were anesthetized with chloral hydrate (500 mg/kg) and sacrificed via cardiac perfusion with 0.1 M PBS, followed by 4% paraformaldehyde (Sigma) in 0.1 M PBS. A 10 mm segment of the spinal cord at the T9 level was dissected, post-fixed in the same fixative for 5 h, and then transferred to 30% sucrose in 0.1 M PBS at 4 °C for 3 days. Samples were embedded in OCT compound for frozen sectioning. Longitudinal or transverse sections were cut at 10–20 μm thickness using a cryostat (CM1850; Leica, Wetzlar, Germany). For molecular analyses, animals were perfused with 0.1 M PBS, and a 10 mm segment of the T9 spinal cord was isolated and stored at − 80 °C until use.

### Gelatin zymography

To assess MMP-9 and MMP-2 activity, gelatin zymography was performed as previously described [10]. Briefly, 50 µg of total protein was loaded onto a Novex 10% zymogram gel (EC61752; Invitrogen, Carlsbad, CA) and separated by electrophoresis at 100 V (19 mA) for 6 h at 4 °C. The gel was incubated in renaturing buffer (2.5% Triton X-100) at room temperature for 30 min to restore gelatinolytic activity. The gel was then incubated in developing buffer (50 mM Tris-HCl, pH 8.5, 0.2 M NaCl, 5 mM CaCl₂, 0.02% Brij-35) at 37 °C for 24 h. After incubation, the gel was stained with 0.5% Coomassie Brilliant Blue and destained with a solution of 40% methanol and 10% acetic acid until clear bands indicating gelatinase activity were visible. These bands were quantified using AlphaImager software (Alpha Innotech Corporation, San Leandro, CA). Three independent experiments were performed, and the data were analyzed statistically.

### RNA isolation and RT-PCR

Total RNA was extracted using the TRIZOL Reagent (Thermo Scientific, Rockford, IL), and 1 µg of RNA was used for first-strand cDNA synthesis with MMLV reverse transcriptase (Thermo) as previously described [11]. The resulting cDNA was subjected to RT-PCR using a thermal cycler (Takara Bio, Japan). Primers for JMJD3, MMP-2, MMP-9, tumor necrosis factor (TNF)-α, interleukin (IL)-6, IL-1β, COX-2, iNOS, MCP-1, MIP-1α, MIP-1β, Gro-α, and GAPDH were synthesized by Genotech (Daejeon, Korea). PCR products were separated using agarose gel electrophoresis (1.5% or 3%) and stained with ethidium bromide. Relative band intensities were measured using AlphaImager software (Alpha Innotech Corporation) and compared to sham control values. Results from three independent experiments were subjected to statistical analysis. The sequences of all primers are presented in Table 1.

**Table 1:** Nucleotide sequences of primers and conditions used for RT-PCR

Target	Primer	Sequence	Annealing temperature (℃)	Cycles
JMJD3	Forward	5’-TTCCTGTTTACCGCTTCG-3’	62	40
	Reverse	5’-CGTTCCACTCATATCGCT-3’		
MMP-2	Forward	5’-ACCATCGCCCATCATCAAGT-3’	55	40
	Reverse	5’-CGAGCAAAAGCATCATCCAC-3’		
MMP-9	Forward	5’-AAAGGTCGCTCGGATGGTTA-3’	55	40
	Reverse	5’-AGGATTGTCTACTGGAGTCGA-3’		
PDGFRα	Forward	5′-CGACTCCAGATGGGAGTTCCC-3′	61	35
	Reverse	5′-TGCCATCCACTTCACAGGCA-3′		
PDGFRβ	Forward	5’-CAACATTTCGAGCACCTTTGT-3’	62	35
	Reverse	5’-AGGGCACTCCGAAGAGGTAA-3’		
IL-6	Forward	5′-AAAATCTGCTCTGGTCTTCTGG-3′	58	30
	Reverse	5′-GGTTTGCCGAGTAGACCTCA-3’		
TNF-α	Forward	5’-CCCAGACCCTCACACTCAGAT-3’	60	33
	Reverse	5’-TTGTCCCTTGAAGAGAACCTG-3’		
COX-2	Forward	5’-CCATGTCAAAACCGTGGTGAATG-3’	58	35
	Reverse	5’-ATGGGAGTTGGGCAGTCATCAG-3’		
IL-1β	Forward	5’-GCAGCTACCTATGTCTTGCCCGTG-3’	58	30
	Reverse	5’-GTCGTTGCTTGTCTCTCCTTGTA-3’		
iNOS	Forward	5’-CTCCATGACTCTCAGCACAGAG-3’	58	30
	Reverse	5’-GCACCGAAGATATCCTCATGAT-3’		
MCP-1	Forward	5’-TCAGCCAGATGCAGTTAACG-3’	55	30
	Reverse	5’-GATCCTCTTGTAGCTCTCCAGC-3’		
MIP-1β	Forward	5’-TCCCACTTCCTGCTGTTTCTCT-3’	60	34
	Reverse	5’-GAATACCACAGCTGGCTTGGA-3’		
Gro-α	Forward	5’-CCGAAGTCATAGCCACACTCAA-3’	65	35
	Reverse	5’-GCAGTCGTCTCTTTCTCCGTTAC-3’		
MIP-1α	Forward	5′-ACTGCCTGCTGCTTCTCCTACA-3′	62	35
	Reverse	5′-AGGAAAATGACACCTGGCTGG-3′		
GAPDH	Forward	5’-TCCCTCAAGATTGTCAGCAA-3’	50	23
	Reverse	5’-AGATCCACAACGGATACATT-3’		

### Western blot

Total protein was isolated using a lysis buffer containing 1% Nonidet P-40, 50 mM Tris (pH 8.0), 150 mM NaCl, 10 mM Na₂P₂O₇, 10 mM NaF, 1 µg/ml aprotinin, 10 µg/ml leupeptin, 1 mM sodium vanadate, and 1 mM phenylmethylsulfonyl fluoride, as previously described [11]. Protein samples (30 µg) were separated by SDS-PAGE (Millipore) and transferred to a nitrocellulose membrane. The membranes were blocked with 5% nonfat skim milk or 5% bovine serum albumin in Tris-buffered saline containing 0.1% Tween-20 for 1 h at room temperature. They were then incubated overnight at 4 °C with primary antibodies listed in Table 2. Secondary antibodies conjugated with horseradish peroxidase (Jackson ImmunoResearch, West Grove, PA) were used to detect the primary antibodies, and immunoreactive bands were visualized using a chemiluminescence kit (Thermo Scientific). Each experiment was performed three times. Band intensities were measured using AlphaImager software (Alpha Innotech Corporation), and background levels were subtracted from optical density values. β-tubulin was used as a loading control.

**Table 2:** Antibody information for Western blot

Antibody	Manufacturer	Catalog number	Dilution used
JMJD3	Abcam	Ab38113	1:1000
H3K27me3	Abcam	Ab6002	1:1000
Histone H3	Cell signaling	9715	1:10000
PDGFRα	Cell signaling	3174	1:1000
PDGFRβ	Cell signaling	3169	1:1000
p-PDGFRα	Cell signaling	3170	1:1000
p-PDGFRβ	Cell signaling	3161	1:1000
ED-1	Bio-Rad	MCA341R	1:1000
iNOS	BD Biosciences	610333	1:1000
COX-2	Cayman chemical	160107	1:1000
Cleaved caspase-3	Cell signaling	9661	1:1000
ZO-1	Thermo Fisher Scientific	40-2200	1:10000
Occludin	Thermo Fisher Scientific	40-4700	1:3000
β-tubulin	Sigma	T4026	1:30000

### Immunohistochemistry

Frozen sections of the spinal cord were immunostained with specific antibodies listed in Table 3. After incubation in blocking solution for 1 h at room temperature, primary antibodies were applied overnight at 4 °C in the same blocking solution. Labeled cells were detected using the ABC method with a Vectastain Kit (Vector Laboratories, Burlingame, CA), and 3,3′-Diaminobenzidine (DAB) was used as the peroxidase substrate. For double labeling, FITC- or Cy3-conjugated secondary antibodies (Jackson ImmunoResearch) were used. Nuclei were stained with DAPI according to the manufacturer’s protocol (Invitrogen). Control samples showed no substrate reaction when the primary antibody was omitted or replaced by a non-immune control antibody.

For quantification of ED-1-, MPO-, and cleaved caspase-3-positive oligodendrocytes (CC1-positive), serial transverse Sect. (20 μm thickness) were collected at 200 μm intervals rostral and caudal to the lesion epicenter. Digital images of MPO- and ED-1-stained tissues were acquired, and fluorescent intensity above the threshold was quantified using MetaMorph software (Molecular device) and averaged. MPO- and ED-1-positive cell intensities were expressed relative to vehicle control. Cleaved caspase-3-positive CC1-positive oligodendrocytes in the white matter (WM) of each section were manually counted and averaged using MetaMorph software. For NF200, serial transverse sections collected every millimeter up to 7 mm rostral and caudal to the lesion site were stained with the NF200 antibody. Axonal densities were measured in pre-selected fields (40 × 40 μm; 1600 μm²) within the vestibulospinal tract (VST) for NF200-positive axons, as described previously [26]. Anatomical landmarks ensured consistent site locations between groups, and axons were manually counted in each field. Results were expressed as a percentage relative to sham control values (100%). All analyses were performed by trained experimenters blinded to experimental conditions.

**Table 3:** Antibody information for immunohistochemistry

Antibody	Manufacturer	Catalog number	Dilution used
p-PDGFRα	Cell signaling	3170	1:100
p-PDGFRβ	Cell signaling	3161	1:100
ED-1	Bio-Rad	MCA341R	1:100
MPO	Dako	A0398	1:100
Cleaved caspase-3	Cell signaling	9661	1:100
NF200	Sigma	N4142	1:100
5-HT	Diasorin	24,430	1:5000
CC1	Abcam	Ab16794	1:100

### TUNEL staining

TUNEL staining was performed on injured spinal cord sections using an ApopTag in situ reagent (Millipore) according to the manufacturer’s instructions. Serial transverse Sect. (20 μm thickness) collected every 200 μm at 1 and 5 days after injury were stained, and a diaminobenzidine substrate kit (Vector Laboratories) was used for peroxidase staining. Control sections were incubated without the TdT enzyme. TUNEL-positive cells in the gray matter (GM) at 1 day (total of 40 sections) and in the white matter (WM) at 5 days (total of 100 sections) after SCI were counted and quantified using a 20× objective. Only cells exhibiting morphological features of nuclear condensation and/or fragmentation in the GM and WM were counted as TUNEL-positive.

### Cell counting of viable ventral motor neurons

To count the number of viable ventral motor neurons (VMNs), serial transverse spinal cord Sect. (20 μm thickness) were collected every 500 μm, extending 8 mm rostrally and caudally from the injury site. VMNs larger than half the area of a sampling square (20 × 20 μm) and located in the lower ventral horn were manually counted and analyzed using MetaMorph software (Molecular Devices), as described previously [11].

### Behavioral tests

The animals were randomly divided into three experimental groups: sham, vehicle, and imatinib-treated, with each group consisting of 10 rats for motor function evaluation. Behavioral tests were conducted on the same day in a randomized order by two investigators blinded to the experimental conditions. The tests included the Basso-Beattie-Bresnahan (BBB) locomotion scale, inclined plane test, grid walk, and footprint analysis, following previously described protocols [26–29]. The BBB scale ranges from 0 to 21 points, systematically assessing hindlimb functional recovery, where 0 represents the absence of hindlimb movement, and 21 represents normal walking ability in rodents. In the inclined plane test, rats were placed on an inclined surface, with either the right or left side up, and the maximum angle at which they could maintain their position without falling for 5 s was recorded and averaged [29].

The grid walk test evaluated the ability to place hindlimbs accurately. Rats were placed on a horizontal runway with elevated metal grid bars (30 cm above the ground) and allowed to explore freely. Video recordings of their movements were analyzed to assess hind paw placement accuracy. Foot slips, defined as hind paws slipping through grid holes, were counted. Total steps and foot slips for each hind paw were recorded within a 1-meter distance, and the mean percentage of foot slips was calculated [27]. For footprint analysis, the rats’ forepaws and hindpaws were dipped in non-toxic red and blue dye, respectively, before walking across a narrow box (1 m long, 7 cm wide). The footprints were scanned and digitized for analysis to assess footprint characteristics [26, 30].

### Axon counting and myelin staining

After the behavioral tests, vehicle- and imatinib-treated rats were perfused at 35 days post-SCI, and frozen sections were prepared as described above. For axonal density analysis, serial transverse sections collected every millimeter, extending 7,000 μm rostrally and caudally from the lesion site, were stained with NF200 antibody. Some sections were also processed for 5-HT staining. The ABC method was used to detect labeled cells using a Vectastain kit (Vector Laboratories). Axonal densities were determined in pre-selected fields (40 × 40 μm, 1,600 μm²) within the ventral and dorsolateral funiculi for NF200-positive axons, as described previously [26, 31]. Serotonin (5-HT) fiber densities were also determined in pre-selected fields (40 × 40 μm, 1,600 μm²) within the ventral horn. The anatomical landmarks ensured consistent site locations across groups, and serotonin fibers and axons were manually counted within each field using MetaMorph software (Molecular Devices). Serotonin fibers and axon counts were expressed as percentages relative to the sham control (100%). For myelin loss assessment, transverse cryosections (2 mm rostral from the lesion site) were incubated overnight in 0.1% Luxol Fast Blue (Solvent Blue 38; Sigma) in acidified 95% ethanol at 60 °C. Differentiation was performed using 0.05% lithium carbonate, as previously described [31]. Digital images of Luxol Fast Blue-stained tissues were obtained, and the intensity above a threshold was quantified using MetaMorph software (Molecular Devices).

### Assessment of lesion volume

Lesion volume in rats subjected to behavioral analysis was measured as described previously [11]. Serial longitudinal Sect. (10 μm thickness) of the spinal cord were prepared, and every 50 μm section was stained with Cresyl Violet acetate. Lesion volume was determined by measuring the cavitation area at the injury epicenter using a low-power objective (1.25×) and calculated using MetaMorph software (Molecular Devices). The area at each longitudinal level was measured, and the total lesion volume was calculated by integrating the sequential areas.

### Statistical analysis

All data are presented as the mean ± standard deviation (SD) or standard errors of the mean (SEM). Statistical comparisons between experimental groups were evaluated using either the unpaired Student’s *t*-test or one-way ANOVA for multiple group comparisons. Repeated measures ANOVA (time vs. treatment) was used for BBB locomotor scale and inclined plane test data. Statistical analysis was performed using SPSS 15.0 (SPSS Science, Chicago, IL), and significance was set at *p* < 0.05.

## Results

### Imatinib inhibits the activation of PDGFRα and β, which are upregulated in vascular endothelial cells after spinal cord injury

**Fig. 1:**
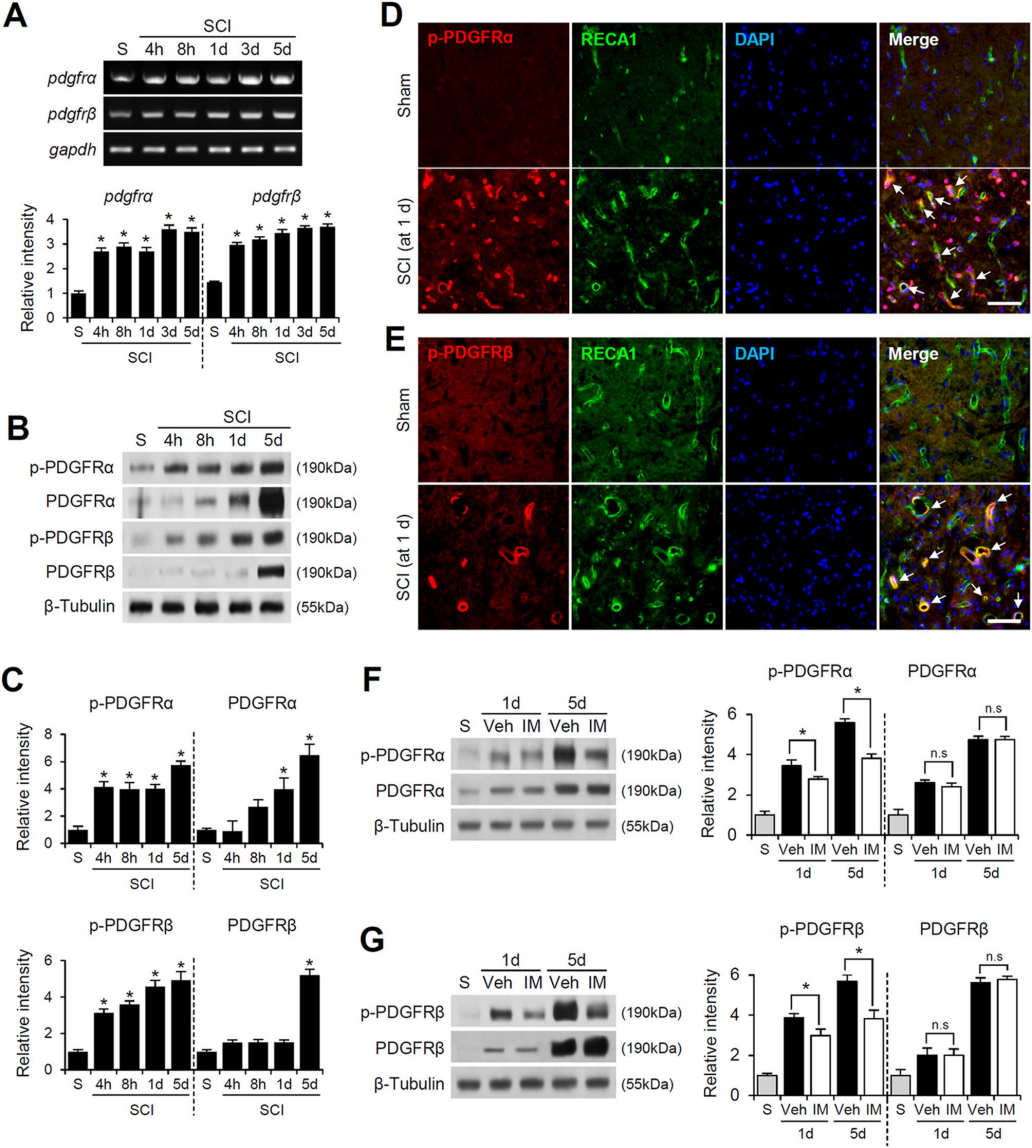
Imatinib inhibits the activation of PDGFRα and β, which are upregulated in vascular endothelial cells after spinal cord injury. (**A**) RT-PCR and densitometric analysis of PDGFRα and β after SCI (mean ± SD, *n* = 5). *p < 0.05 vs. Sham (one-way ANOVA). (**B**) Western blot of p-PDGFRα, PDGFRα, p-PDGFRβ, PDGFRβ and β-Tubulin after SCI. (**C**) Densitometric analysis of Western blot (mean ± SD, *n* = 5). *p < 0.05 vs. Sham (one-way ANOVA). (**D**) Double immunofluorescence staining of p-PDGFRα with RECA1(blood vessel) in injured spinal cord at 1 d after SCI. Scale bar, 50 μm. (**E**) Double immunofluorescence staining of p-PDGFRβ with RECA1 in injured spinal cord at 1 d after SCI. Scale bar, 50 μm (*n* = 3). (**F**-**G**) Western blot and densitometric analysis for p-PDGFRα, PDGFRα, p-PDGFRβ, PDGFRβ and β-Tubulin at 1 d and 5 d after SCI. (mean ± SD, *n* = 5). *p < 0.05 vs. Vehicle (one-way ANOVA)

To investigate the role of PDGFR signaling in blood-spinal cord barrier (BSCB) disruption after SCI, we first examined changes in PDGFR expression and activity using RT-PCR, Western blotting, and immunofluorescence staining. The mRNA and protein levels of PDGFRα and PDGFRβ were significantly upregulated, peaking at 5 days post-SCI compared to the sham group (Fig. 1A, B). Similarly, the levels of phosphorylated (p)-PDGFRα and p-PDGFRβ, the activated forms of these receptors, were markedly increased, peaked at 5 days post-SCI (Fig. 1B and C). Immunofluorescence labeling further revealed that both p-PDGFRα and p-PDGFRβ co-localized with RECA1-positive endothelial cells in the injured spinal cord as early on day post-SCI (Fig. 1D and E). These results indicate that PDGFR expression and activation are upregulated in vascular endothelial cells following SCI.

To investigate the effect of imatinib, a tyrosine kinase inhibitor, on PDGFR activation after SCI, we assessed the levels of phosphorylated PDGFRα and PDGFRβ (p-PDGFRα and p-PDGFRβ) using Western blotting. The data showed that imatinib did not alter the total levels of PDGFRα and PDGFRβ (Fig. 1F and G). Densitometric analysis confirmed no significant difference in PDGFRα and PDGFRβ levels between the vehicle- and imatinib-treated groups (Fig. 1F and G). However, imatinib significantly reduced the levels of p-PDGFRα and p-PDGFRβ at 1 and 5 days post-SCI, indicating that imatinib effectively suppresses PDGFR phosphorylation without affecting total protein expression (Fig. 1F and G).

### Imatinib also inhibits PDGFRα and β activation in OGD/R-induced bEnd.3 cells

**Fig. 2:**
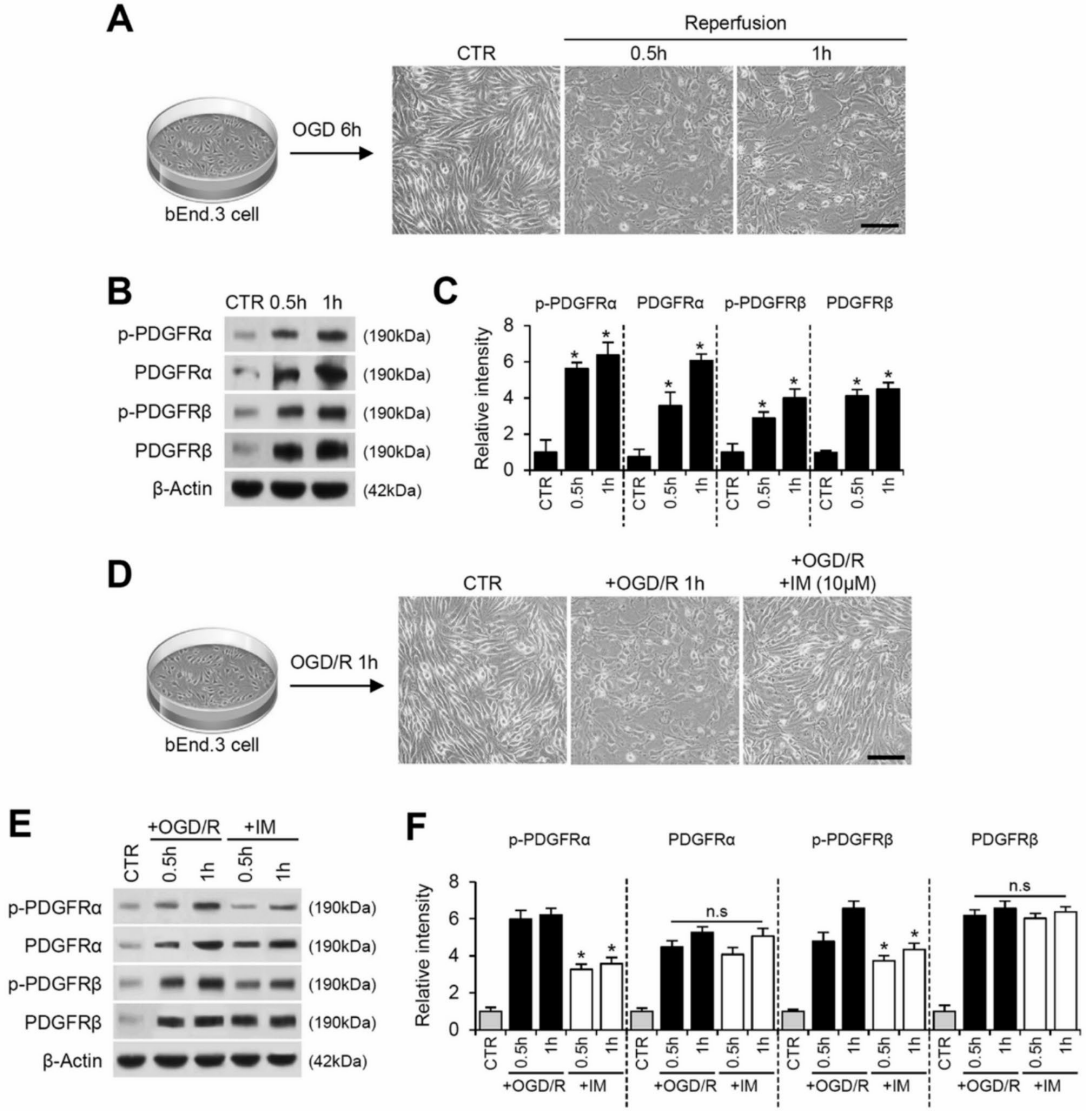
Imatinib also inhibits PDGFRα and β activation in OGD/R-induced bEnd.3 cells. (**A**) Schematic illustration of experimental protocol. Cells were exposed to OGD for 6 h, followed by reperfusion for 0.5–1 h. Photographs show morphological change of bEnd.3 cells. scale bar, 50 μm. (**B**) Western blot for p-PDGFRα, PDGFRα, p-PDGFRβ, PDGFRβ and β-actin. (**C**) Densitometric analysis of Western blot (mean ± SD, *n* = 5). *p < 0.05 vs. CTR (one-way ANOVA). (**D**) Cells were exposed to OGD for 6 h, followed by reperfusion for 1 h. Photographs show morphological change of bEnd.3 cells. scale bar, 50 μm. (**E**) Western blot for p-PDGFRα, PDGFRα, p-PDGFRβ, PDGFRβ and β-actin in OGD/R-induced bEnd.3 cells treated with imatinib. (**F**) Densitometric analysis of western blot (mean ± SD, *n* = 5). *p < 0.05 vs. +OGD/R (one-way ANOVA)

Since PDGFR signaling was activated in blood vessels after SCI, we examined the effect of imatinib on PDGFR signaling in endothelial cells using an in vitro model of oxygen-glucose deprivation/reoxygenation (OGD/R)-induced bEnd.3 cells. Control bEnd.3 cells (CTR) exhibited a distinct and integrated morphology, whereas OGD/R-induced bEnd.3 cells displayed a loss of their characteristic spindle-shaped appearance and prominent cell retraction (Fig. 2A). Concurrently, both PDGFRα and PDGFRβ, as well as their phosphorylated forms, were upregulated in OGD/R-induced bEnd.3 cells (Fig. 2B and C). Imatinib treatment, however, notably improved the morphology of OGD/R-induced bEnd.3 cells (Fig. 2D) and significantly inhibited OGD/R-induced p-PDGFRα and p-PDGFRβ expression (Fig. 2E and F). Consistent with in vivo findings, the total levels of PDGFRα and PDGFRβ in bEnd.3 cells were not affected by imatinib treatment (Fig. 2E and F). Collectively, these findings suggest that while imatinib does not alter PDGFR expression, it specifically inhibits PDGFR activation in vascular endothelial cells after SCI.

### Imatinib prevents BSCB disruption and tight junction breakdown by inhibiting MMP-9 expression and activation after SCI

**Fig. 3:**
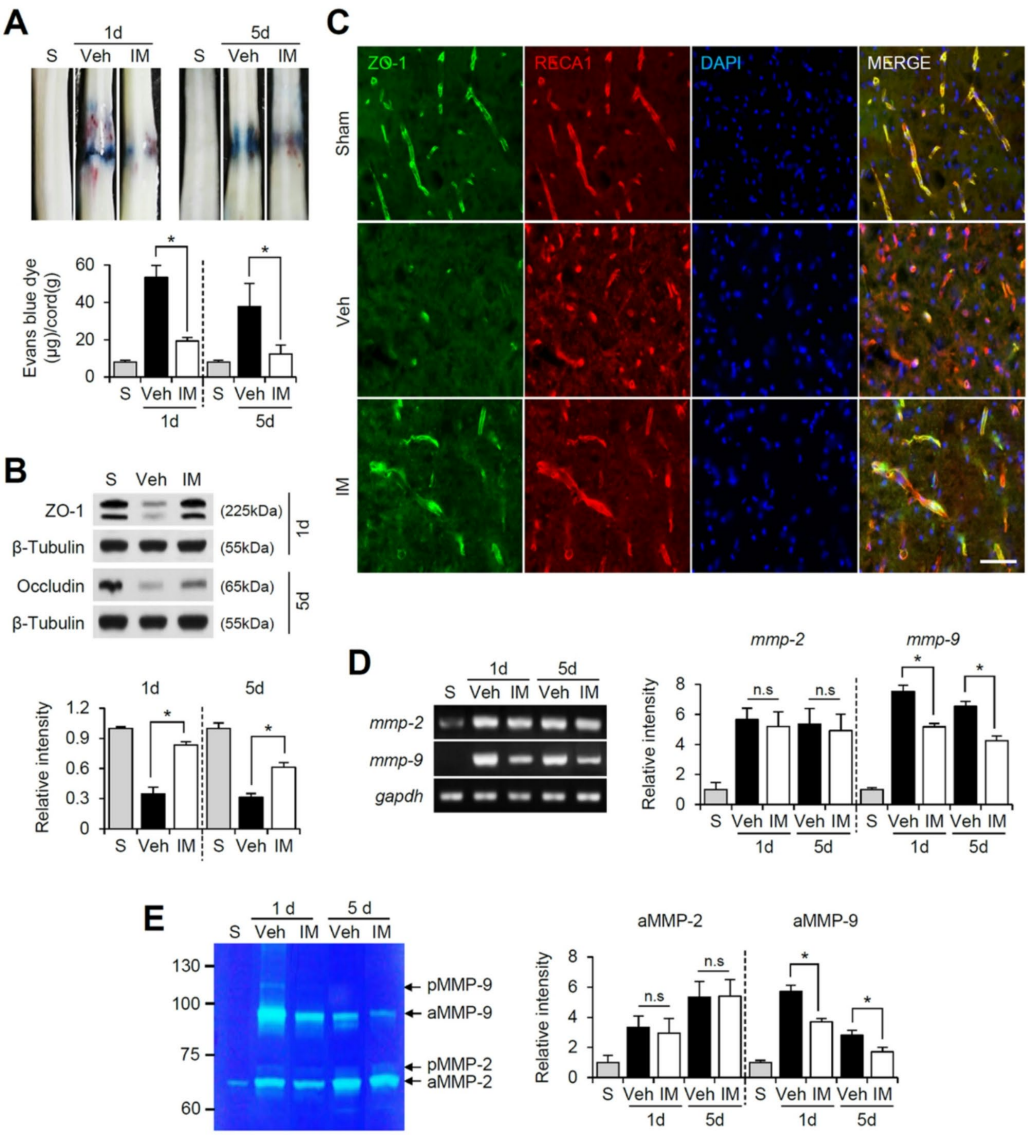
Imatinib prevents BSCB disruption and tight junction breakdown by inhibiting MMP-9 expression and activation after SCI. (**A**) Representative spinal cord showing Evans blue dye permeabilized into moderately injured spinal cord at 1 d and 5 d after SCI. Quantification of the amount of Evans blue at 1 d and 5 d after injury (mean ± SEM, *n* = 5). *p < 0.05 vs. Vehicle (unpaired t-test). (**B**) Western blot for ZO-1 (at 1 d) and occludin (at 5 d) after SCI and densitometric analysis of western blot (mean ± SD, *n* = 5). (**C**) Double immunofluorescence staining of ZO-1 with RECA1(blood vessel) in injured spinal cord at 1 d after SCI. Scale bar, 50 μm. (**D**) RT-PCR and densitometirc analysis of mmp-2 and − 9 mRNA (mean ± SD, *n* = 5). (**E**) Gelatin zymography and densitometric analysis of zymography (mean ± SD, *n* = 5). *p < 0.05 vs. Vehicle (one-way ANOVA). pMMP-2, pro MMP-2; aMMP-2, active MMP-2; pMMP-9, pro MMP-9; aMMP-9, active MMP-9

Given that SCI induces PDGFRα and PDGFRβ activation in vascular endothelial cells, and imatinib effectively inhibits PDGFR signaling after SCI (Figs. 1 and 2), we hypothesized that imatinib would reduce BSCB permeability after SCI. To test this hypothesis, we examined the effect of imatinib on BSCB permeability at 1 and 5 days post-SCI using the Evans blue assay. As shown in Fig. 3A, SCI led to a significant increase in Evans blue dye extravasation compared to uninjured sham controls, indicating BSCB leakage. Imatinib treatment, however, significantly reduced Evans blue dye extravasation at both 1 and 5 days post-injury compared to vehicle controls (Fig. 3A). Notably, imatinib reduced the Evans blue dye extravasation to near-sham levels by 5 days post-SCI. Next, we investigated whether imatinib preserves tight junction (TJ) integrity following SCI using Western blot analysis. Imatinib significantly mitigated the decrease in ZO-1 and occludin levels at 1 and 5 days post-SCI compared to vehicle controls (Fig. 3B). In addition, double immunofluorescence staining for ZO-1 and RECA-1 showed that the fragmentation of capillary blood vessel was increased after SCI and ZO-1 immunoreactivity was decreased upon injury compared with the sham control. This vascular fragmentation is indicative of disrupted BSCB integrity, as structural breakdown of microvessels is often associated with tight junction loss. However, imatinib treatment attenuated the fragmentation of the capillary blood vessels and ZO-1 loss (Fig. 3C).

Given that imatinib preserved TJ integrity, we further examined whether imatinib inhibits the expression and activation of matrix metalloproteinases (MMPs), which are known contributors to BSCB disruption after SCI. RT-PCR analysis revealed that mRNA levels of MMP-2 and MMP-9 increased post-SCI. However, imatinib significantly suppressed MMP-9 mRNA expression at 1 and 5 days post-SCI, while MMP-2 mRNA expression remained unchanged (Fig. 3D). Both MMP-2 and MMP-9 activities using gelatin zymography were then examined. SCI resulted in marked increase in MMP-2 and MMP-9 activities, as evidenced by bands corresponding to their active forms (Fig. 3E). Imatinib treatment significantly reduced the active form of MMP-9 at 1 and 5 days post-SCI compared to vehicle controls, while MMP-2 activity was unaffected (Fig. 3E). These findings indicate that imatinib-mediated inhibition of PDGFR signaling preserves TJ integrity by specifically reducing MMP-9 expression and activation, thereby effectively preventing BSCB disruption following SCI.

### Imatinib alleviates tight junction breakdown by inhibiting JMJD3 expression and activation after SCI and OGD/R injury

**Fig. 4:**
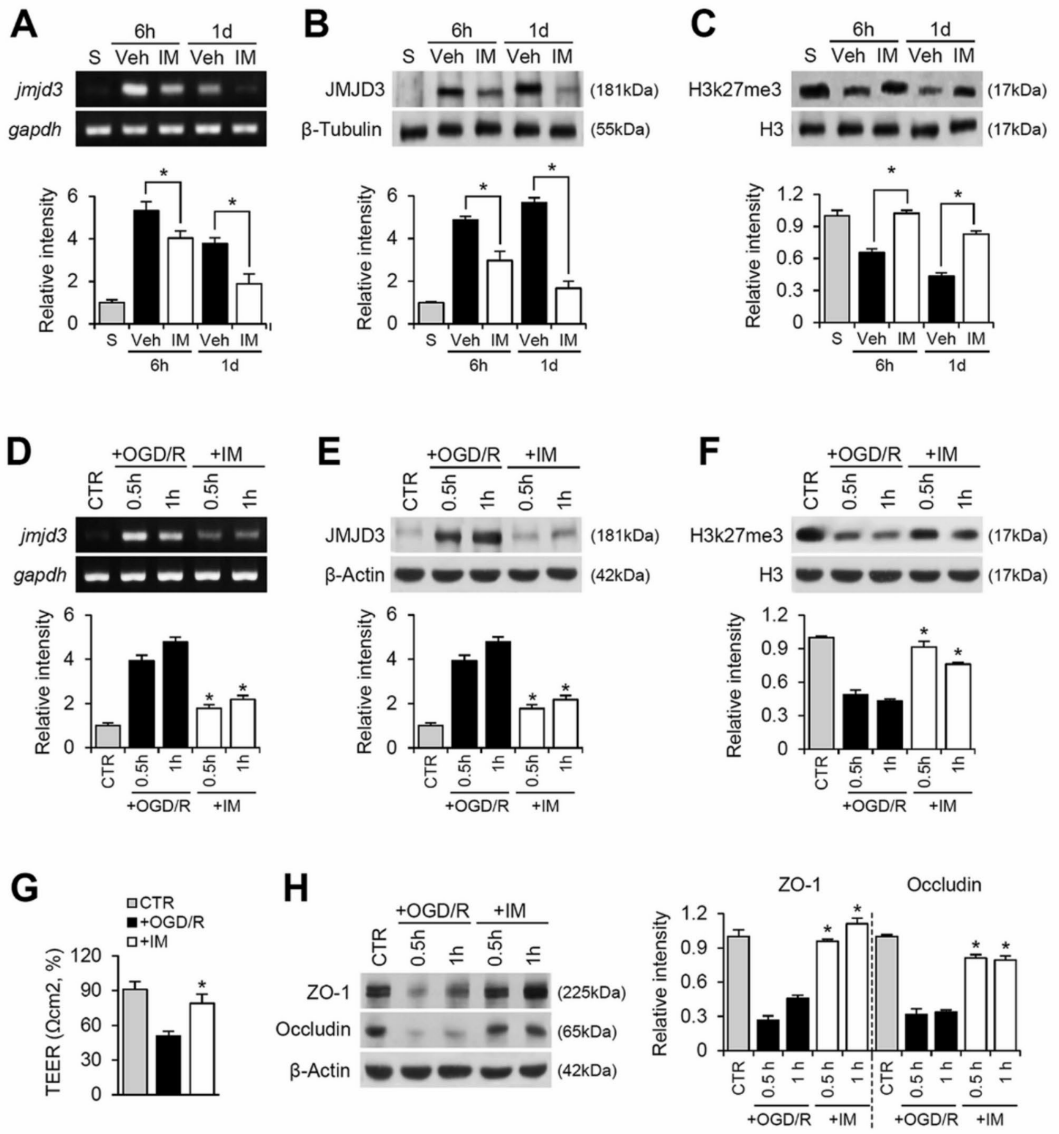
Imatinib alleviates tight junction breakdown by inhibiting JMJD3 expression and activation after SCI and in OGD/R-induced bEnd.3 cells. (**A**) RT-PCR for jmjd3 after SCI and densitometric analysis of RT-PCR (mean ± SD, *n* = 5). Western blot for (**B**) JMJD3 and (**C**) H3k27me3 after SCI. Densitometric analysis of Western blot (mean ± SD, *n* = 5). *p < 0.05 vs. Vehicle (one-way ANOVA). (**D**) RT-PCR for jmjd3 and densitometric of RT-PCR (mean ± SD, *n* = 5). (**E**) Western blot for JMJD3 and densitometric analysis (mean ± SD, *n* = 5). (**F**) Western blot for H3k27me3/H3 and densitometric analysis (mean ± SD, *n* = 5). (**G**) TEER (mean ± SD, *n* = 5). (**H**) Western blot for ZO-1 and occludin. Densitometric analysis of Western blot (mean ± SD, *n* = 5). *p < 0.05 vs. +OGD/R (one-way ANOVA)

Our previous study demonstrated that SCI-induced BSCB disruption is associated with increased JMJD3 expression and activation [13]. To determine whether JMJD3 is regulated by PDGFR signaling, we examined JMJD3 expression and activity in response to PDGFR inhibition using imatinib after SCI and in OGD/R-induced bEnd.3 cells. RT-PCR and Western blot analyses revealed that JMJD3 mRNA and protein levels increased after injury compared to the sham control (Fig. 4A and B). However, imatinib treatment significantly reduced JMJD3 mRNA expression at 6 h and 1 day post-SCI (Fig. 4A, IM). Similarly, JMJD3 protein levels were significantly lower in the imatinib-treated group compared to vehicle controls (Fig. 4B). Since JMJD3 acts as a histone demethylase that removes methyl groups from H3K27me3, we next assessed its activity by examining H3K27me3 levels via Western blotting. The results showed a reduction in H3K27me3 levels post-injury compared to the sham control. Imatinib treatment, however, significantly restored H3K27me3 levels compared to vehicle control (Fig. 4C). These findings suggest that JMJD3 expression and activation are at least partially regulated by PDGFR signaling after SCI.

JMJD3 mRNA levels were also markedly increased at 0.5 and 1 h after OGD/R injury (Fig. 4D). However, imatinib treatment significantly inhibited JMJD3 mRNA expression at these time points (Fig. 4D). Western blot analysis further confirmed that imatinib treatment significantly reduced JMJD3 protein levels in OGD/R-injured cells (Fig. 4E). We then examined the effect of imatinib on H3K27me3 levels. After OGD/R injury, H3K27me3 levels were significantly reduced (Fig. 4F, +OGD/R). However, imatinib treatment significantly increased H3K27me3 levels compared to vehicle controls post-OGD/R injury (Fig. 4F, +IM). These results suggest that blocking PDGFR signaling effectively inhibits JMJD3 expression and activation in vascular endothelial cells after OGD/R injury. To further confirm the role of PDGFR signaling in BSCB disruption, we examined the effect of imatinib on endothelial cell permeability using transendothelial electrical resistance (TEER) measurements and Western blot analysis of TJ proteins. TEER values were significantly lower in OGD/R-injured bEnd.3 cells compared to untreated controls, indicating increased permeability in OGD/R-injured bEnd.3 cells (Fig. 4G, +OGD/R). Imatinib treatment, however, substantially prevented the TEER decrease caused by OGD/R injury (Fig. 4G, +IM). Consistent with these findings, the expression of TJ proteins ZO-1 and occludin was significantly reduced in bEnd.3 cells subjected to OGD/R injury (Fig. 4H, +OGD/R), reflecting a disruption of TJ integrity. However, imatinib treatment during OGD/R injury significantly mitigated this decrease in ZO-1 and occludin expression (Fig. 4H, +IM), thereby preserving TJ integrity. These findings suggest that imatinib protects the BSCB by maintaining TJ integrity through the inhibition of PDGFR-mediated JMJD3 expression and activation in vascular endothelial cells during OGD/R injury.

### PDGFR-JMJD3 axis mediates tight junction breakdown after SCI and OGD/R injury

**Fig. 5:**
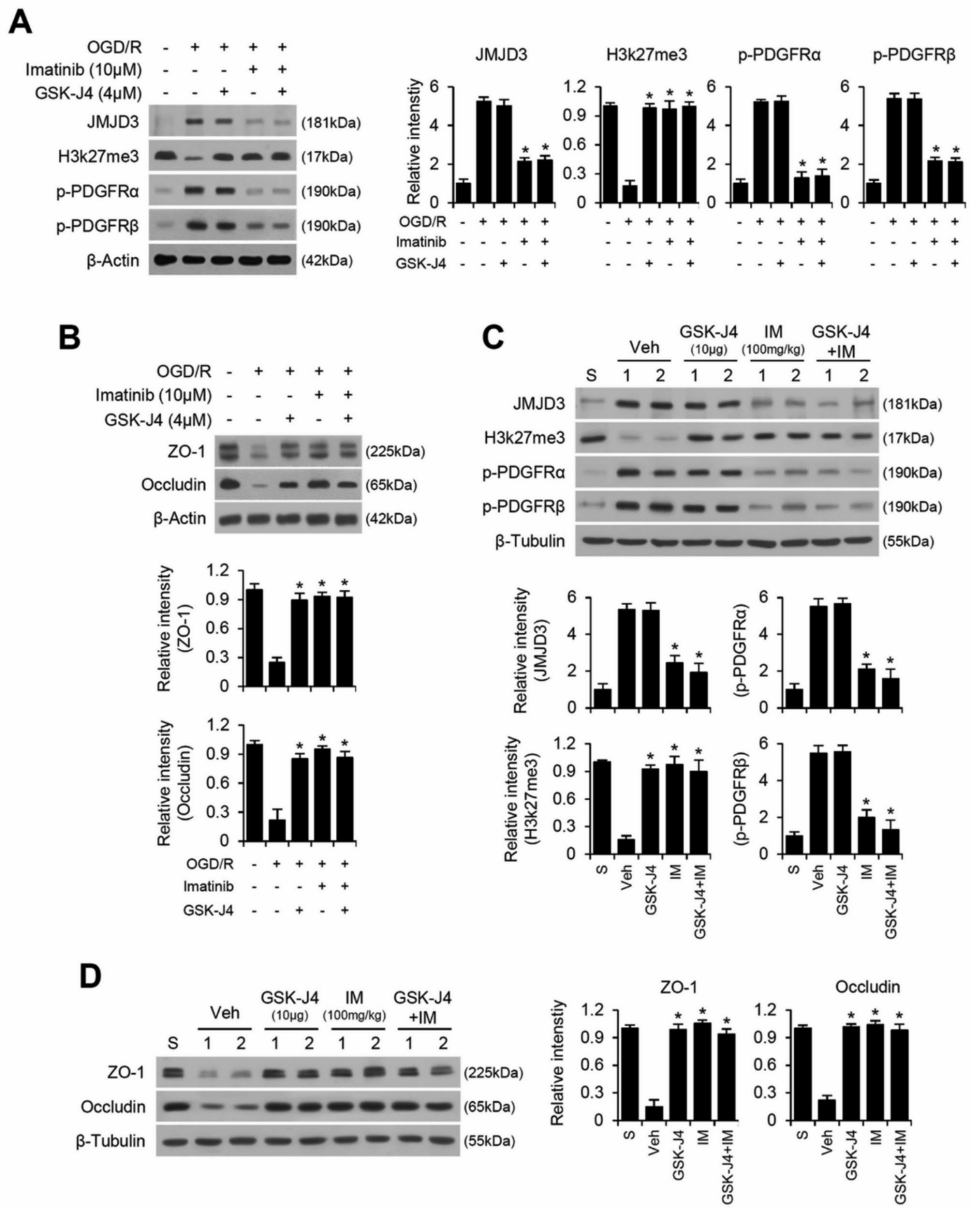
PDGFR-JMJD3 axis mediates tight junction breakdown after SCI and OGD/R injury. Western blot and densitometric analysis for (**A**) JMJD3, H3k27me3, p-PDGFRα, p-PDGFRβ, (**B**) ZO-1 and occludin in imatinib and/or GSK-J4 treated End.3 cells subjected to 1 h of OGD/R injury (mean ± SD, *n* = 5). *p < 0.05 vs. +OGD/R (one-way ANOVA). Western blot and densitometric analysis for (**C**) JMJD3, H3k27me3, p-PDGFRα, p-PDGFRβ, (**D**) ZO-1 and occludin in injured spinal cord at 1 d after SCI (mean ± SD, *n* = 5). *p < 0.05 vs. Vehicle (one-way ANOVA)

To determine whether imatinib protects against BSCB disruption through PDGFR-JMJD3 signaling axis after SCI, we examined JMJD3 expression, H3K27me3 levels, and PDGFRα/β phosphorylation in an OGD/R injury model of endothelial cells. As shown in Fig. 5A, OGD/R injury significantly increased PDGFRα/β phosphorylation and JMJD3 expression, while decreasing H3K27me3 levels. Treatment with GSK-J4 increased H3K27me3 levels without affecting JMJD3 expression, confirming that GSK-J4 inhibits JMJD3 enzymatic activity rather than its expression. Furthermore, imatinib significantly reduced PDGFRα/β phosphorylation, decreased JMJD3 expression, and increased H3K27me3 levels in OGD/R-induced bEnd.3 cells, suggesting that PDGFR activation is a key upstream regulator of JMJD3 expression and activity. Supporting this hypothesis, co-treatment with imatinib and GSK-J4 did not produce additional effects beyond those observed with imatinib alone, indicating that PDGFR likely acts upstream of JMJD3 in this signaling pathway in endothelial cells. Next, we assessed the expression of TJ proteins in bEnd.3 cells subjected to OGD/R injury. Treatment with GSK-J4, imatinib, or their combination effectively prevented TJ breakdown (Fig. 5B). Notably, co-treatment did not enhance the protective effect beyond that of imatinib alone, further suggesting that PDGFR inhibition suppresses JMJD3 expression and activity, thereby promoting BSCB integrity (Fig. 5B).

To validate these findings, we analyzed spinal cord tissues collected from injured animals treated with the respective drugs. Treatment with GSK-J4 significantly increased H3K27me3 levels without altering JMJD3 expression, confirming that GSK-J4 specifically inhibits JMJD3 enzymatic activity (Fig. 5C). Furthermore, imatinib treatment effectively suppressed PDGFRα/β phosphorylation, reduced JMJD3 expression, and subsequently increased H3K27me3 levels, suggesting that PDGFR activation also regulates JMJD3 expression and activity (Fig. 5C). Consistent with the in vitro data, co-treatment with imatinib and GSK-J4 did not yield additive effects compared to imatinib alone, further supporting the hierarchical relationship wherein PDGFR signaling regulates JMJD3 expression after SCI (Fig. 5C). Finally, to assess the functional impact of PDGFR-JMJD3 signaling on BSCB integrity after SCI, we examined the expression of TJ proteins ZO-1 and occludin in injured spinal cord tissues treated with the respective drugs (Fig. 5D). Treatment with GSK-J4, imatinib, or their combination effectively preserved TJ integrity and prevented BSCB disruption one day post-SCI. Collectively, these findings establish PDGFR as a critical upstream regulator of JMJD3-mediated BSCB disruption after SCI.

### Imatinib inhibits neutrophil and macrophage infiltration and reduces chemokines and cytokines expression after SCI

**Fig. 6:**
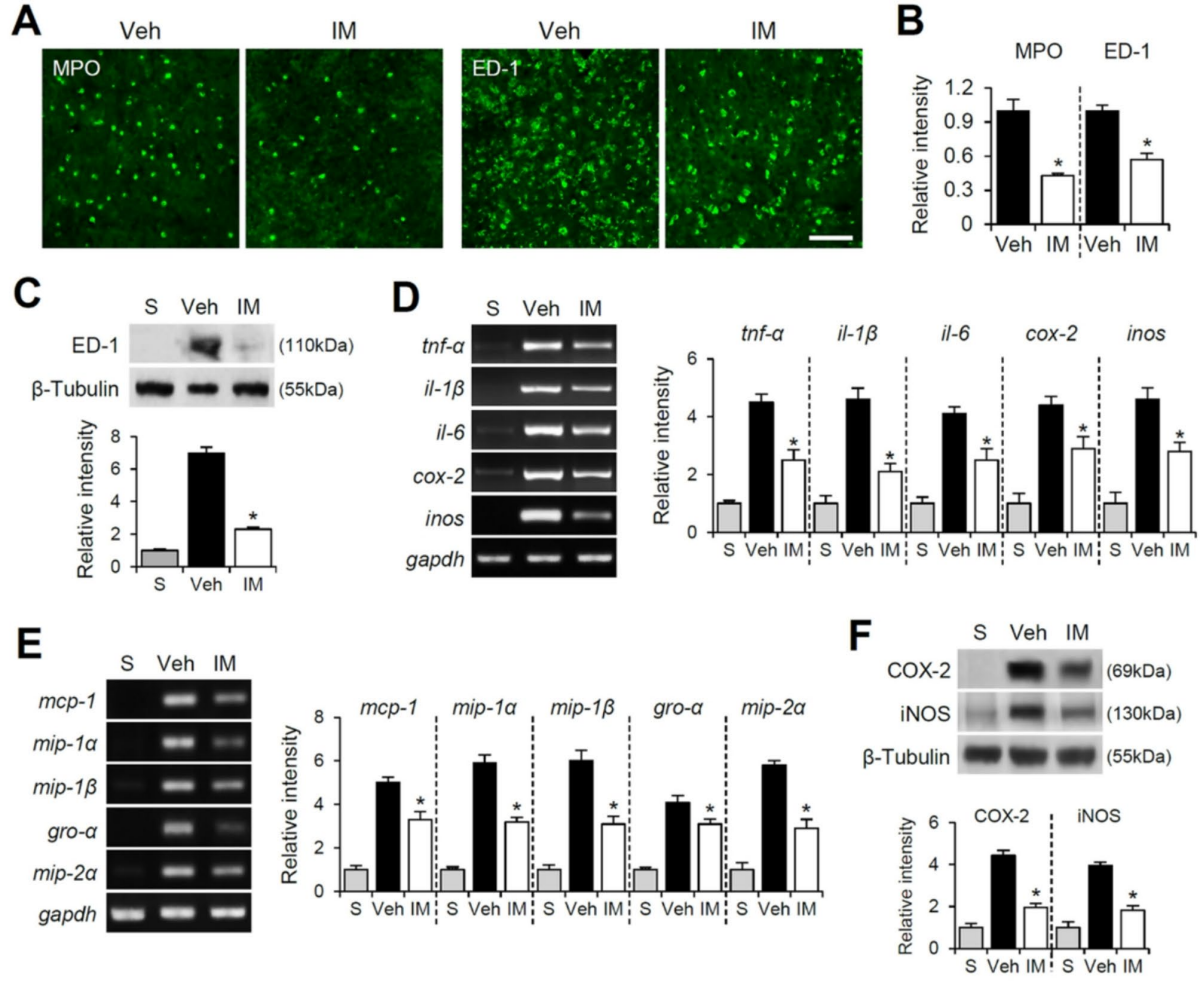
Imatinib inhibits neutrophil and macrophage infiltration and reduces chemokines and cytokines expression after SCI. After SCI, blood infiltration was assessed by measuring the fluorescent intensity of MPO or ED-1 immunoreactive area at 1 and 5 d or Western blot for ED-1 at 5 d after injury as described in the Materials & methods section. (**A**) Representative photographs at 1500 and 2000 μm rostral to lesion epicenter showed MPO-labeled neutrophils and ED-1-labeled macrophages in the dorsal column of injured spinal tissues (cross section) injected with and without imatinib. Scale bar, 50 μm. (**B**) Relative fluorescent intensity of MPO- and ED-1-positive cells (mean ± SD, *n* = 5). *p < 0.05 vs. Vehicle (unpaired t-test). (**C**) Western blot and densitometric analysis of ED-1 at 5 d after injury (mean ± SD, *n* = 5). (**D**) RT-PCR and densitometric analysis for cytokines (*tnf-α at 2 h*,* il-1β* and *il-6* at 6 h, *cox-2* and *inos* at 1 d) (mean ± SD, *n* = 5). (**E**) RT-PCR and densitometric analysis for chemokines (*mcp-1*,* mip-1α* and *mip-1β* at 6 h, *gro-α* and *mip-2α* at 2 h) (mean ± SD, *n* = 5). (**F**) Western blot and densitometric analysis of COX-2 and iNOS at 1 d after SCI (mean ± SD, *n* = 5). *p < 0.05 vs. Vehicle (one-way ANOVA)

To investigate the effect of imatinib treatment on blood cell infiltration following SCI, we performed immunofluorescence labeling and Western blotting using antibodies against MPO and ED-1, markers for neutrophils and macrophages, respectively. Immunofluorescence staining revealed numerous MPO-positive cells at 1 day and ED-1-positive cells at 5 days post-SCI in the dorsal column of the injured spinal cord. However, imatinib treatment significantly reduced the number of infiltrating MPO- and ED-1-positive cells compared to vehicle controls (Fig. 6A and B). Western blot analysis further confirmed a marked increase in ED-1 protein levels after SCI, which was significantly attenuated by imatinib treatment (Fig. 6C). Next, we evaluated the effect of imatinib on the expression of inflammatory cytokines and chemokines following SCI using RT-PCR and Western blotting. Imatinib significantly inhibited the mRNA expression of TNF-α and IL-1β at 2 h post-SCI, as well as IL-6, COX-2, and iNOS at 6 h post-SCI (Fig. 6D). Furthermore, imatinib reduced the mRNA expression of chemokines, including MCP-1, MIP-1α, MIP-1β, and Gro-α, at 2 h post-SCI (Fig. 6E). Additionally, imatinib treatment significantly reduced the protein levels of COX-2 and iNOS at 1 day post-SCI (Fig. 6F). These findings suggest that imatinib effectively reduces the infiltration of neutrophils and macrophages into the injured spinal cord and suppresses the expression of pro-inflammatory chemokines and cytokines following SCI.

### Imatinib inhibits neuronal and oligodendrocyte cell death after SCI

**Fig. 7:**
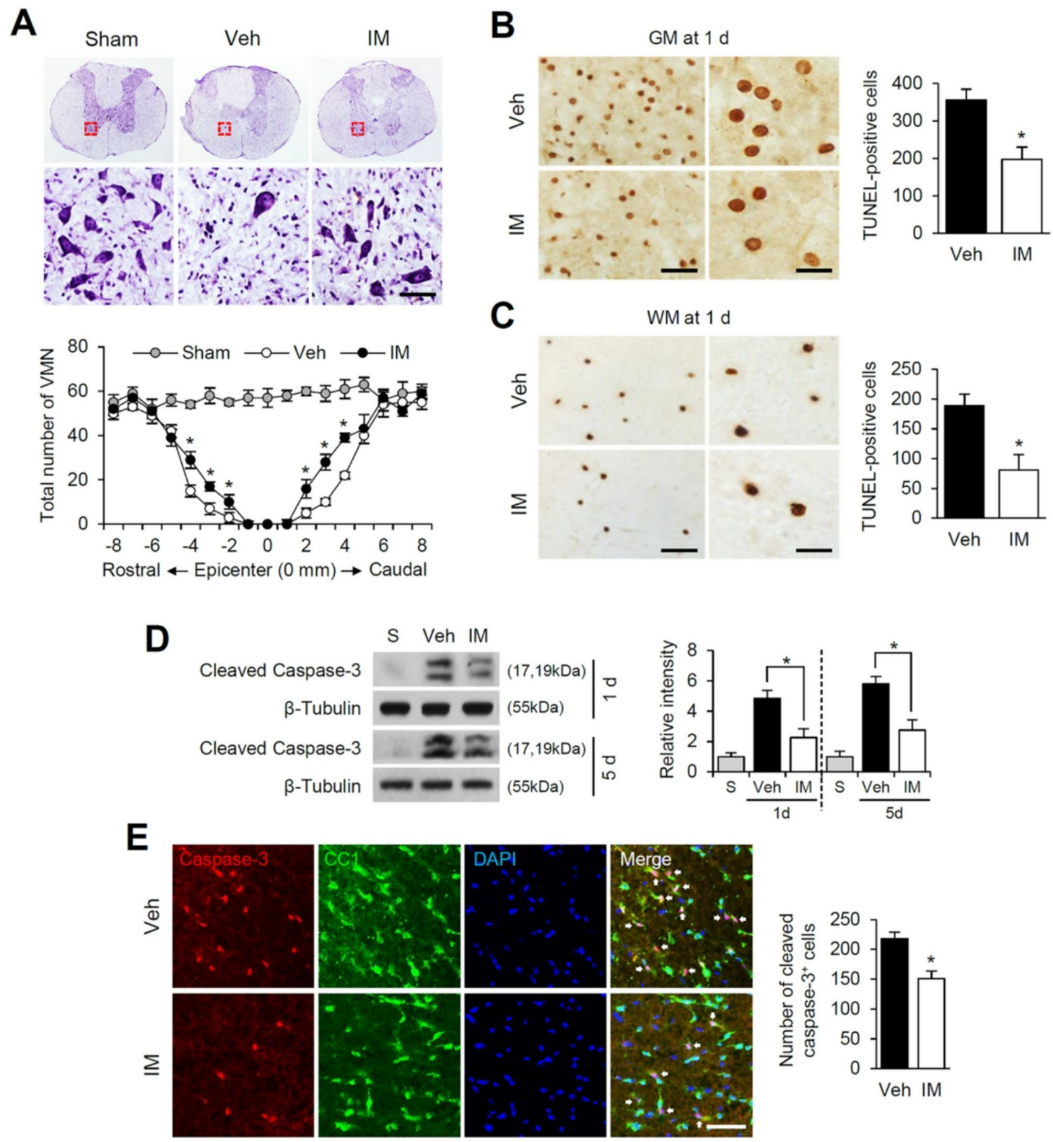
Imatinib inhibits neuronal and oligodendrocyte cell death after SCI. (**A**) Representative Cresyl violet staining showing ventral horn of spinal cord at 3 mm rostral from lesion site at 1 d. Scale bar, 50 μm. The spatial pattern of the number of VMN (mean ± SD, *n* = 3). *p < 0.05 vs. Vehicle (unpaired t-test). (**B**) Representative TUNEL staining in the GM of the spinal cord at 2 mm rostral from lesion site at 1 d and quantitative analysis of TUNEL-positive cells at 1 d (mean ± SD, *n* = 3). *p < 0.05 vs. Vehicle (unpaired t-test). Right panels show high-power views. Scale bars, 20 μm. (**C**) Representative TUNEL staining in the WM at 7 mm rostral from lesion site at 5 d and quantitative analysis of TUNEL-positive cells (mean ± SD, *n* = 3). *p < 0.05 vs. Vehicle (unpaired t-test). Right panels show high-power views. Scale bars, 20 μm. (**D**) Western blot and densitometirc analysis of cleaved caspase-3 at 1 d and 5 d after injury (mean ± SD, *n* = 5). *p < 0.05 vs. Vehicle (one-way ANOVA). (**E**) Immunohistochemical analysis of cleaved caspase-3 and CC1. Double labeling shows that oligodendrocytes in the WM were positive for cleaved caspse-3 after SCI (arrow). Scale bar, 50 μm. Quantitative analysis of cleaved caspase-3-positive cells (mean ± SD, *n* = 3). *p < 0.05 vs. Vehicle (unpaired t-test) (*n* = 3)

To investigate the neuroprotective effects of imatinib following SCI, we performed Cresyl violet staining to assess motor neuron survival in the ventral horn at 1 day post-SCI. As shown in Fig. 7A, a significant loss of ventral motor neurons (VMNs) was observed in the lesion area after SCI, consistent with previous reports [32]. However, imatinib treatment markedly reduced VMN loss both rostral and caudal to the lesion epicenter compared to the vehicle-treated group (Fig. 7A). TUNEL staining revealed that most TUNEL-positive cells were located near and within the lesion area in the GM at 1 day post-SCI (Fig. 7B) and predominantly outside the lesion area in the WM at 5 days post-SCI (Fig. 7C). Imatinib treatment significantly decreased the number of TUNEL-positive neurons in the GM at 1 day (Fig. 7B) and TUNEL-positive oligodendrocytes in the WM at 5 days post-SCI (Fig. 7C) compared to the vehicle-treated controls. Western blot analysis showed that imatinib significantly reduced levels of cleaved caspase-3, a marker of apoptosis, at both 1 and 5 days post-SCI compared to vehicle controls (Fig. 7D). Double immunofluorescence staining revealed that cleaved caspase-3-positive cells in the WM at 5 days post-SCI were CC1-positive oligodendrocytes (Fig. 7E). Furthermore, imatinib treatment substantially reduced the number of activated caspase-3-positive cells in the WM at 5 days post-SCI (Fig. 7E). These findings demonstrate that imatinib exerts neuroprotective effects by reducing apoptosis of both neurons and oligodendrocytes following SCI.

### Imatinib attenuates axon and myelin loss and improves functional recovery after SCI

**Fig. 8:**
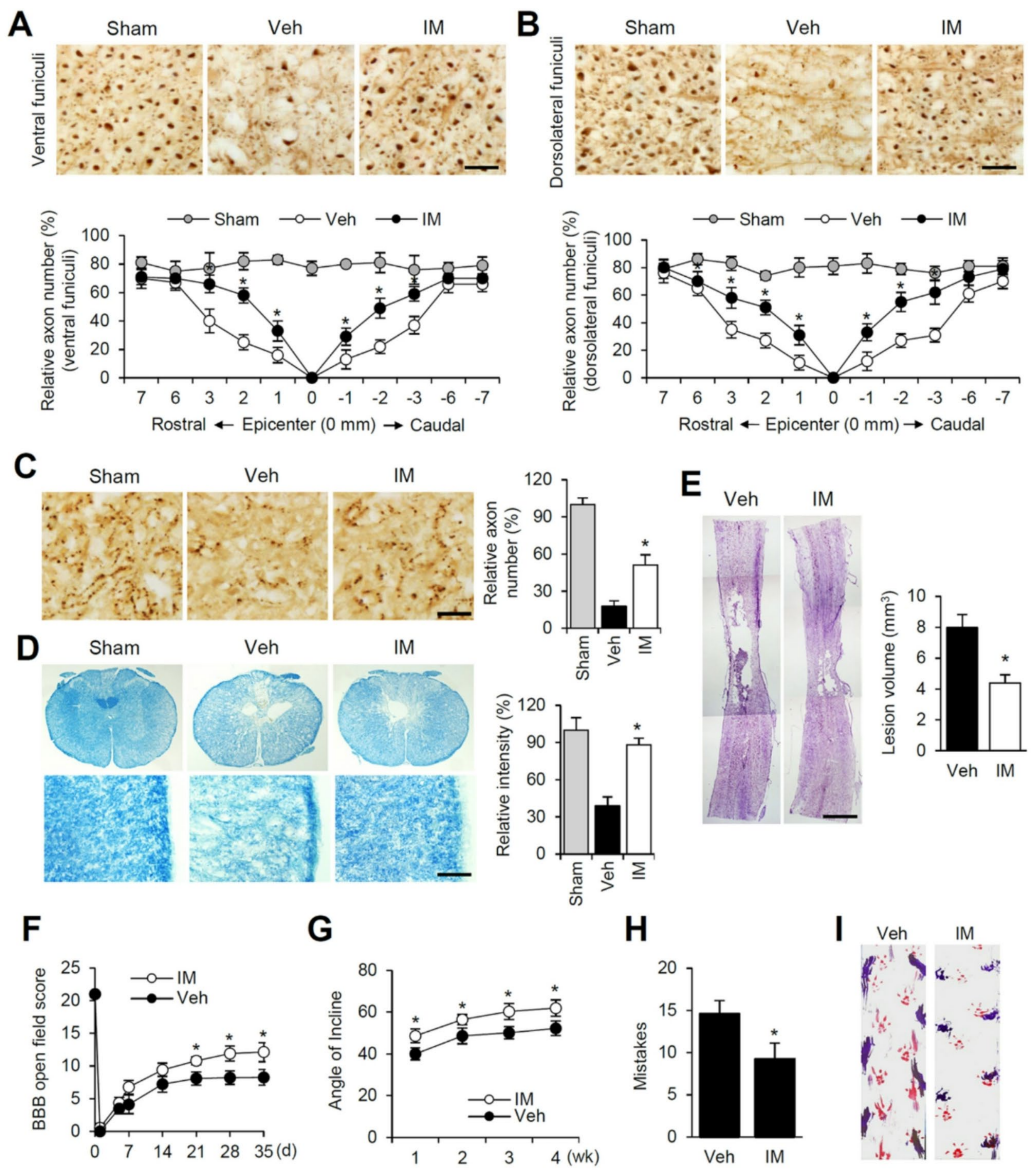
Imatinib attenuates axon and myelin loss and improves functional recovery after SCI. (**A**) Representative photographs of NF200-positive axons in ventral funiculus of spinal cord. Scale bars, 50 μm. Quantitative analysis of NF200-positive axons (mean ± SD, *n* = 5). *p < 0.05 vs. Vehicle (unpaired t-test). (**B**) Representative photographs of NF200-positive axons in dorsolateral funiculus of spinal cord and quantitative analysis (mean ± SD, *n* = 5). *p < 0.05 vs. Vehicle (unpaired t-test). (**C**) Representative photographs of 5-HT-positive axon in ventral horn areas in Sect. 3 mm caudal to the lesion site. Scale bars, 50 μm. Quantitative analysis of 5-HT (mean ± SD, *n* = 5). *p < 0.05 vs. Vehicle (unpaired t-test). (**D**) Transverse sections (lateral funiculus) selected from 2 mm rostral to the lesion site were processed for Luxol fast blue staining. Note that the extent of myelin loss was attenuated by imatinib treatment compared to the vehicle control. Scale bar, 100 μm. Quantitative analysis of Luxol fast blue intensity (mean ± SD, *n* = 5). *p < 0.05 vs. Vehicle (unpaired t-test). (**E**) Representative spinal cord tissues (1.2 mm from the dorsal surface) showing cavitation in the lesion site and quantitative analysis at 35 d after injury. Scale bar, 1 mm. Data are presented as mean ± SD (*n* = 5). *p < 0.05 vs. Vehicle (unpaired t-test). (**F**) BBB scores, (**G**) Inclined plane test, (**H**) Grid walk test, and (**I**) Foot print analysis of vehicle and imatinib-treated groups after SCI. Behavior data are presented as mean ± SEM (*n* = 10). *p < 0.05 vs. Vehicle (one-way repeated measured ANOVA)

Traumatic SCI induces immediate mechanical damage, followed by a complex secondary cascade of degenerative processes, including axonal degeneration and demyelination, ultimately leading to progressive tissue loss [13]. To assess axonal preservation density, we performed immunostaining using NF200 and 5-HT antibodies. In the sham control group, axons were densely packed, with homogeneous NF200-positive axonal distributions in the ventral and dorsolateral funiculi (Fig. 8A and B, Sham). However, axonal density was significantly reduced in the injured spinal cords of the vehicle-treated animals (Fig. 8A and B, Veh). In contrast, the imatinib-treated group exhibited a substantially greater number of NF200-positive axons in both the ventral and dorsolateral funiculi (Fig. 8A and B, IM). Furthermore, imatinib-treated group showed a higher abundance of 5-HT-positive serotonergic axons in the ventral horn compared to the vehicle-treated animals (Fig. 8C), indicating that imatinib alleviates axonal loss after SCI. To evaluate myelin preservation, Luxol fast blue staining was performed. Significant myelin loss was observed near the lesion site in the vehicle-treated group at 35 days post-injury compared to the sham control group (Fig. 8D, Veh). However, imatinib treatment markedly preserved myelin in the same region (Fig. 8D, IM). Furthermore, total lesion volume was significantly reduced in the imatinib-treated group at 35 days post-injury compared to the vehicle-treated controls (Fig. 8E).

Next, we investigated whether imatinib treatment improves functional deficits following SCI. Imatinib (100 mg/kg, i.p.) was administered immediately, 2 h, and 8 h after injury, and subsequently once daily for 14 days. As shown in Fig. 8F, imatinib significantly improved hindlimb locomotor function from 14 to 35 days post-injury compared to the vehicle-treated group. Additionally, imatinib-treated rats displayed a significantly greater angle of incline from 1 to 4 weeks post-injury (Fig. 8G). At 35 days post-injury, hindlimb coordination was evaluated using the grid walk test. Imatinib-treated rats made significantly fewer footfall errors compared to vehicle-treated controls (Fig. 8H). Footprint analysis further revealed improved forelimb-hindlimb coordination and reduced toe dragging in imatinib-treated rats, as evidenced by more consistent stepping patterns and minimal ink drag marks from the hindlimbs (Fig. 8I). In contrast, vehicle-treated rats exhibited irregular dorsal stepping and pronounced toe dragging. These findings collectively demonstrate that imatinib significantly enhances functional recovery, reduces tissue damage, and preserves axonal and myelin integrity following SCI.

## Discussion

In this study, we identified a novel neuroprotective mechanism of imatinib that enhances BSCB integrity and improves functional outcomes after SCI. Specifically, we elucidated the pathway through which imatinib preserves BSCB integrity by inhibiting PDGFR activation. Our findings demonstrate that PDGFR activation regulates JMJD3 expression and activity, which in turn controls the expression of (MMPs. These observations were consistent across both in vivo SCI model and in vitro OGD/R-induced injury model using endothelial cells.

Imatinib was administered intraperitoneally at 100 mg/kg immediately after SCI, followed by additional doses at 8 h post-injury and once daily for 14 days. Preliminary dose-finding experiments with 50, 100, and 200 mg/kg revealed that 100 mg/kg significantly reduced Evans blue extravasation and MMP-9 activities compared to vehicle-treated controls (see Supplementray Fig. 1). This dose was selected as optimal, as it provided therapeutic efficacy without significant adverse effects or weight loss. It is worth noting that short-term imatinib treatment for BSCB protection differs from its chronic use in oncology. Further studies are necessary to define the optimal dosage and treatment duration for SCI. GSK-J4, a selective JMJD3 inhibitor, was delivered via intraspinal injection at 10 µg/rat following SCI. Dose-response experiments (3, 10, and 30 µg) indicated that 10 µg was the minimal effective dose, as it significantly affected TJ breakdown and produced effects comparable to the 30 µg dose (see Supplementray Fig. 2). To minimize variability and potential toxicity, 10 µg/rat was used in subsequent experiments. While intrathecal or intraperitoneal administration is common in SCI models, intraspinal injection offers direct tissue targeting and potentially more localized effects, making it advantageous for SCI research.

BSCB disruption is a critical contributor to secondary injury after SCI, facilitating blood cell infiltration, inflammation, and neuronal apoptosis [33]. Currently, no approved drugs effectively preserve BSCB integrity. Imatinib, a tyrosine kinase inhibitor primarily used in chronic myeloid leukemia and gastrointestinal stromal tumors [18], has shown neuroprotective effects in various CNS disorders, including multiple sclerosis, ischemic stroke, brain hemorrhage, and SCI [17, 20, 22, 34, 35]. Our study demonstrates that imatinib inhibits PDGFR signaling to preserve BSCB integrity after SCI. Notably, PDGFR activation was prominently observed in vascular structures in injured spinal cords, especially in endothelial cells (Fig. 1), and in OGD/R-treated endothelial cells in vitro (Fig. 2). Imatinib significantly suppressed PDGFR phosphorylation in both models, confirming its role as a targetable mediator of BSCB breakdown. These findings align with previous studies implicating PDGFR signaling in B-BB disruption across multiple CNS pathologies. For instance, PDGFR-α activation contributes to B-BB impairment via p38 MAPK in intracerebral hemorrhage [19], while PDGFRβ inhibition increases B-BB permeability and exacerbates brain edema in cerebral ischemia [21]. Our data suggest that similar mechanisms underlie BSCB breakdown after SCI, where endothelial PDGFR activation likely initiates secondary injury processes such as immune cell infiltration and neuronal degeneration. These results highlight PDGFR as a central mediator of vascular pathology in SCI and elucidate how imatinib exerts protective effects. While imatinib targets multiple kinases, our data support the conclusion that its therapeutic efficacy in SCI is primarily driven by PDGFR inhibition.

Understanding the molecular mechanisms regulating BSCB integrity is essential for developing effective treatments. MMPs are established mediators of barrier disruption in CNS injuries [6, 9, 31, 36]. Their degradation of extracellular matrix and TJ proteins increases vascular permeability, exacerbating secondary damage. Our previous studies demonstrated that JMJD3, a histone demethylase, is recruited to the promoters of MMP-2, MMP-3, and MMP-9 genes in endothelial cells, where it directly regulates their expression via epigenetic modifications [11, 13, 14]. This recruitment results in the activation of MMP transcription, which plays a critical role in barrier breakdown. Although the functions and mechanisms of JMJD3 in inflammation, macrophage polarization, and neural injury are relatively well characterized [37, 38], Its role as a regulator of blood-spinal cord barrier (BSCB) disruption remains largely unexplored outside of our own work, and aside from the research conducted by our group, there is minimal existing knowledge about JMJD3’s involvement in BSCB regulation. Our current study provides the first mechanistic evidence linking PDGFR signaling to JMJD3-mediated MMP expression in SCI. We observed marked PDGFR activation in vascular structures post-SCI, and its inhibition by imatinib reduced both JMJD3 and MMP expression. These findings suggest that PDGFR activation initiates a cascade that drives JMJD3-dependent MMP induction, contributing to BSCB breakdown. To further validate this pathway, we co-administered GSK-J4 and imatinib following SCI. Notably, the combination did not provide additive protection compared to imatinib alone (Fig. 5), indicating that JMJD3 inhibition does not enhance BSCB protection beyond PDGFR blockade. This strongly supports a model in which PDGFR activation functions upstream of JMJD3, driving MMP-mediated BSCB disruption. The PDGFR–JMJD3–MMP axis described here represents a novel signaling pathway in SCI, and to our knowledge, this is the first report linking PDGFR signaling to epigenetic regulation of barrier integrity via JMJD3.

Although we did not directly investigate the intermediate signaling molecules between PDGFR and JMJD3, previous studies suggest involvement of pathways such as NF-κB, STATs, TGF-β, and HIF-1 [38, 39]. For example, NF-κB-mediated JMJD3 activation in microglia promotes neurodegeneration in diseases like ALS, Parkinson’s, and Alzheimer’s diseases [39, 40, 41], and our prior work demonstrated NF-κB–JMJD3–MMP signaling in BSCB disruption after SCI [13]. Similarly, JMJD3 is regulated by STAT1 and STAT3 in response to inflammatory stimuli [42], and PDGFR activation has been shown to stimulate NF-κB and STAT pathways in various inflammatory contexts [16, 42–44]. Together, our findings and previous literature support the plausibility that PDGFR regulates JMJD3 via NF-κB and/or STAT3. Future studies are warranted to dissect the precise signaling intermediates, especially in endothelial cells. Additionally, recent reports indicate that imatinib has antioxidant properties in SCI [34]. Reactive oxygen species (ROS), including nitric oxide and peroxynitrite, are known to induce apoptosis and inflammatory gene expression (e.g., COX-2, iNOS) [45]. ROS also contribute to B-BB permeability and disruption [46]. While our study did not assess oxidative stress, the antioxidant effects of imatinib deserve further exploration in the context of BSCB integrity.

## Conclusion

**Fig. 9:**
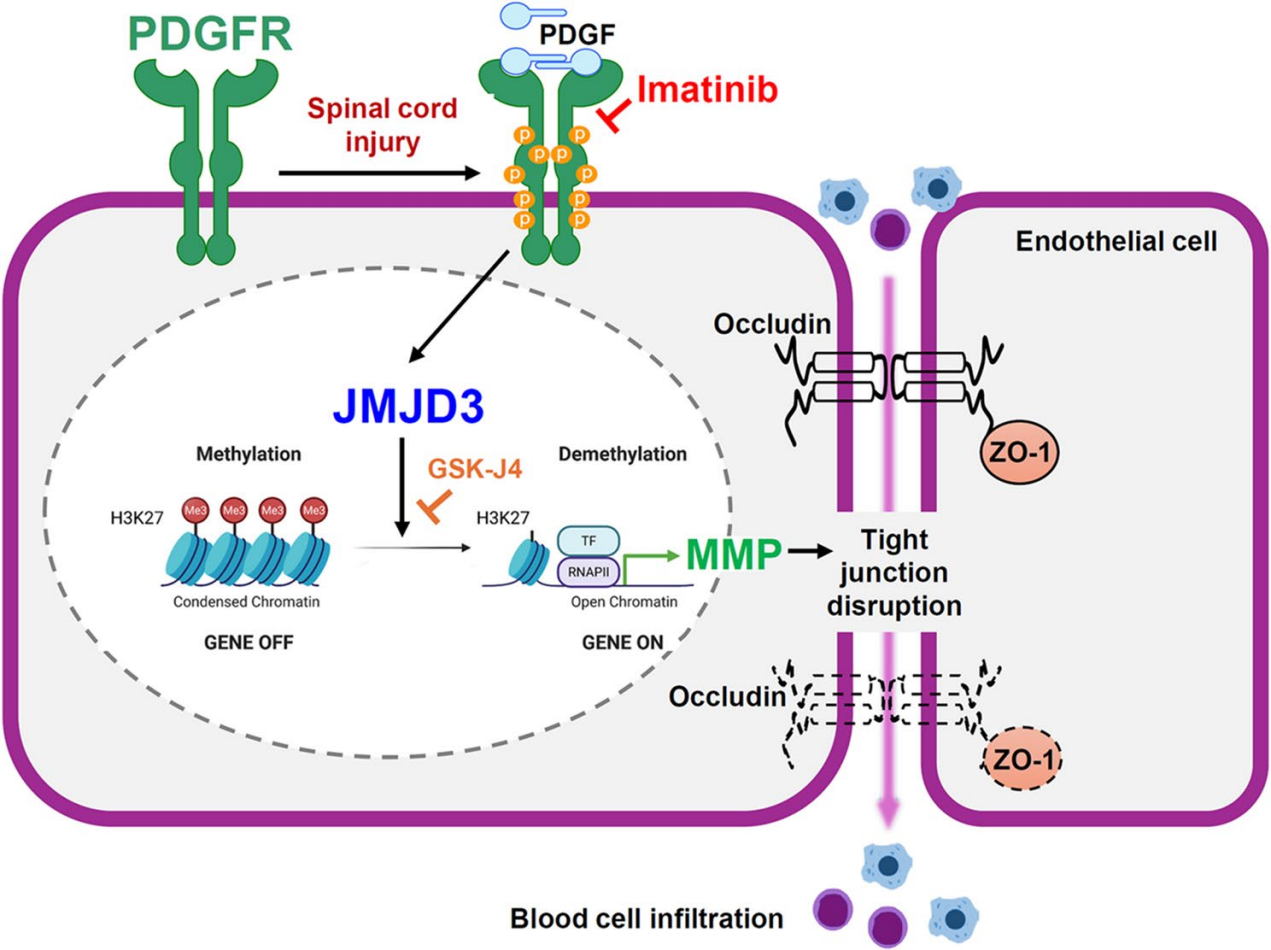
Schematic diagram

Our study demonstrates that PDGFR activation contributes to BSCB disruption after SCI by regulating JMJD3 expression and activity. Imatinib inhibits PDGFR signaling, thereby preserving BSCB integrity and reducing secondary damage (Fig. 9). This is the first study, to our knowledge, to elucidate a direct mechanistic link between PDGFR signaling and JMJD3-mediated MMP activation in SCI. These findings highlight imatinib as a promising therapeutic agent for preserving BSCB integrity and improving neurological outcomes following SCI.

## Electronic supplementary material

Below is the link to the electronic supplementary material.

Supplementary Material 1

## Data Availability

No datasets were generated or analysed during the current study.

## Abbreviations

DAB: 3,3’-Diaminobenzidine
BBB: Basso-Beattie-Bresnahan
B-BB: Blood-brain barrier
BSCB: Blood-spinal cord barrier
CNS: Central nervous system
CTR: Control bEnd.3 cells
DMEM: Dulbecco’s Modified Eagle Medium
DMSO: Dimethyl sulfoxide
GM: Gray matter
IL: Interleukin
IM: Imatinib
i.p.: Intraperitoneal
MMPs: Matrix metalloproteinases
OGD: Oxygen-glucose deprivation
OGD/R: OGD/reperfusion
P: Phosphorylated
PDGFR: Platelet-derived growth factor receptor
ROS: Reactive oxygen species
SCI: Spinal cord injury
SD: Standard deviation
SEM: Standard errors of the mean
TJ: Tight junction
TEER: Transendothelial electrical resistance
TNF: Tumor necrosis factor
Veh: Vehicle-treated group
VMNs: Ventral motor neurons
VST: Vestibulospinal tract
WM: White matter
