# Supplementary material for: Imatinib prevents blood-spinal cord barrier disruption by inhibiting PDGFR-mediated JMJD3 expression and activation after spinal cord injury
Source: Fluids Barriers CNS. 2025 Jul 16;22:76. doi: 10.1186/s12987-025-00690-5 (PMC12269138; doi:10.1186/s12987-025-00690-5)
Supplement: Supplementary file 1 — Supplementary Material 1 [file 12987_2025_690_MOESM1_ESM.docx]

**Supplementary Data**

**
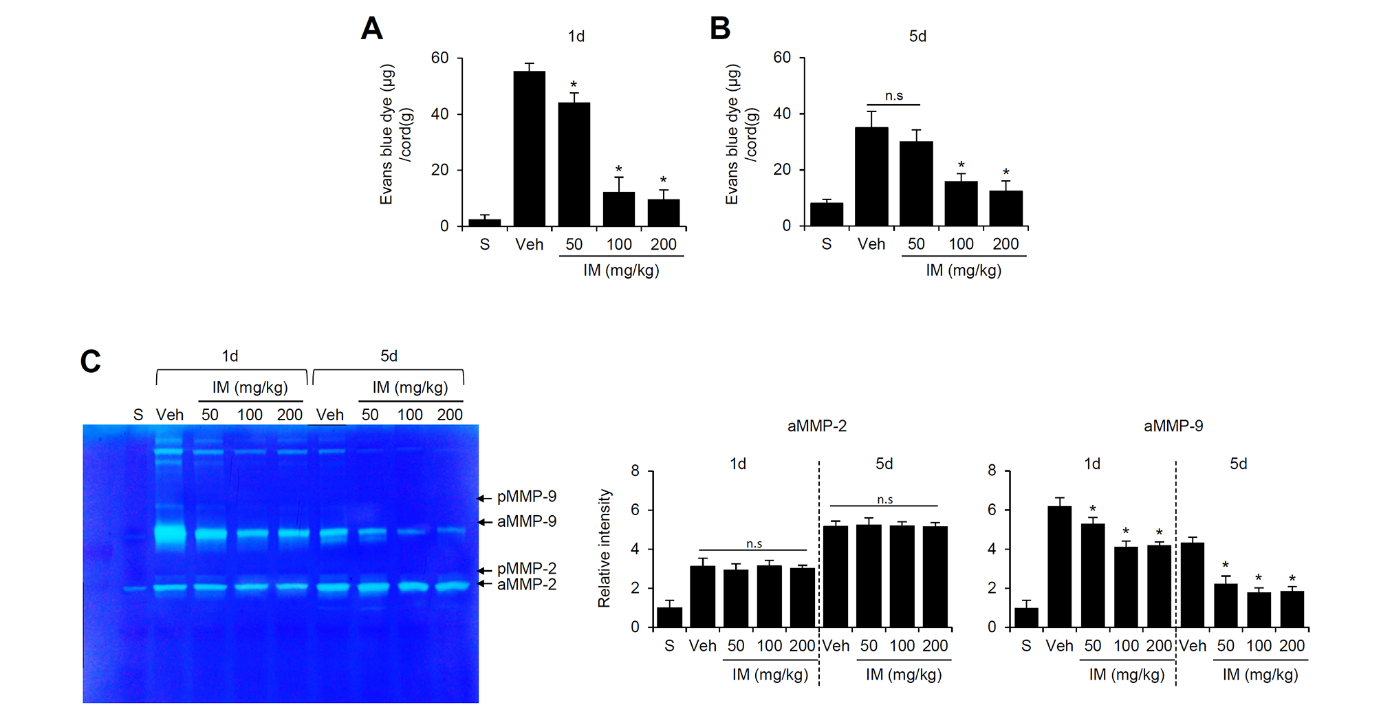
**

**Supplementary Figure 1. Determination of optimal effective concentration of imatinib after SCI.** Quantification of Evans blue dye extravasation at (A) 1 day and (B) 5 days after SCI following treatment with different doses of imatinib (50, 100, and 200 mg/kg, i.p injection) (mean ± SEM, n = 3 per group). **p < 0.05* vs. Vehicle (one-way ANOVA with Tukey’s post hoc test). (C) Representative gelatin zymography for MMP-2 and MMP-9 activity in spinal cord tissue at 1 day and 5 days after SCI with different doses of imatinib. Densitometric analysis of active MMP-2 and MMP-9 bands (mean ± SD, n = 3). **p < 0.05* vs. Vehicle (one-way ANOVA with Tukey’s post hoc test). pMMP-2, pro MMP-2; aMMP-2, active MMP-2; pMMP-9, pro MMP-9; aMMP-9, active MMP-9.


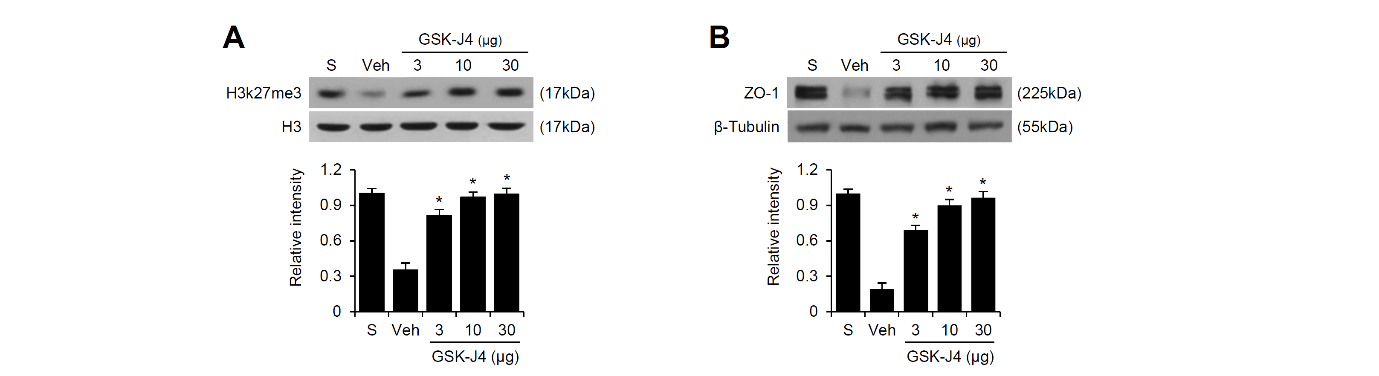


**Supplementary Figure 2. Determination of optimal effective concentration of GSK-J4 after SCI.** Western blot and densitometric analysis of H3K27me3 (A) and ZO-1 (B) in the injured spinal cord at 1 day after SCI following treatment with different doses of GSK-J4 (3, 10, and 30 µg/rat, intraspinal injection) (mean ± SD, n = 3). **p < 0.05* vs. Vehicle (one-way ANOVA).
